# Assessment of Spatial Variability across Multiple Pollutants in Auckland, New Zealand

**DOI:** 10.3390/ijerph16091567

**Published:** 2019-05-05

**Authors:** Ian Longley, Brett Tunno, Elizabeth Somervell, Sam Edwards, Gustavo Olivares, Sally Gray, Guy Coulson, Leah Cambal, Courtney Roper, Lauren Chubb, Jane E. Clougherty

**Affiliations:** 1 National Institute of Water and Atmospheric Research (NIWA), Private Bag 99940, Newmarket, Auckland 1149, New Zealand; ian.longley@niwa.co.nz (I.L.); Elizabeth.Somervell@niwa.co.nz (E.S.); Sam.Edwards@niwa.co.nz (S.E.); Gustavo.Olivares@niwa.co.nz (G.O.); sally.gray@niwa.co.nz (S.G.); Guy.Coulson@niwa.co.nz (G.C.); 2 University of Pittsburgh Graduate School of Public Health, Department of Environmental and Occupational Health, Pittsburgh, PA 15219, USA; btunno@verizon.net (B.T.); ltipton86@gmail.com (L.C.); clr56@pitt.edu (C.R.); lgc4@pitt.edu (L.C.); 3 Drexel University Dornsife School of Public Health, Department of Environmental and Occupational Health, Philadelphia, PA 19104, USA

**Keywords:** particle composition, spatial saturation, source identification, shipping emissions

## Abstract

Spatial saturation studies using source-specific chemical tracers are commonly used to examine intra-urban variation in exposures and source impacts, for epidemiology and policy purposes. Most such studies, however, has been performed in North America and Europe, with substantial regional combustion-source contributions. In contrast, Auckland, New Zealand, a large western city, is relatively isolated in the south Pacific, with minimal impact from long-range combustion sources. However, fluctuating wind patterns, complex terrain, and an adjacent major port complicate pollution patterns within the central business district (CBD). We monitored multiple pollutants (fine particulate matter (PM_2.5_), black carbon (BC), elemental composition, organic diesel tracers (polycyclic aromatic hydrocarbons (PAHs), hopanes, steranes), and nitrogen dioxide (NO_2_)) at 12 sites across the ~5 km^2^ CBD during autumn 2014, to capture spatial variation in traffic, diesel, and proximity to the port. PM_2.5_ concentrations varied 2.5-fold and NO_2_ concentrations 2.9-fold across the CBD, though constituents varied more dramatically. The highest-concentration constituent was sodium (Na), a distinct non-combustion-related tracer for sea salt (µ = 197.8 ng/m^3^ (SD = 163.1 ng/m^3^)). BC, often used as a diesel-emissions tracer, varied more than five-fold across sites. Vanadium (V), higher near the ports, varied more than 40-fold across sites. Concentrations of most combustion-related constituents were higher near heavy traffic, truck, or bus activity, and near the port. Wind speed modified absolute concentrations, and wind direction modified spatial patterns in concentrations (i.e., ports impacts were more notable with winds from the northeast).

## 1. Introduction

Saturation monitoring and modeling using source-specific chemical tracers is now a common approach for examining intra-urban variation in pollution exposures and source impacts [1,2,3,4]—both for air quality management and epidemiology purposes—given consistent evidence of air pollution impacts on respiratory, cardiovascular, and other health outcomes [5,6,7,8,9,10,11,12]. Most such studies, however, have assessed mean spatial variation across a broad metropolitan area, rather than focusing on fine-scale variation within a very small area, or during periods of peak population exposures. Furthermore, most intra-urban studies have emphasized combustion-related pollutants distributed across the urban area (e.g., traffic-related emissions), rather than that of a major adjacent source (e.g., a major port), nor included combustion-related organic tracers of source emissions. Finally, most urban saturation studies have been performed in North America or Europe, where substantial regional pollution and long-range pollution transport can obscure local source impacts, and complicate interpretations of spatial patterns in pollution [13,14,15].

In contrast to most large cities in North America and Europe, Auckland, New Zealand, home to 1.42 million people (2013 census), is relatively isolated in the south Pacific Ocean, with only occasional impacts from the nearest upwind combustion sources in Australia, 2100 km to the west [4]. As a result, background PM_2.5_ concentrations are relatively low (averaging approximately 4 µg/m^3^ at regulatory monitors on the urban peripheries). Importantly, this isolation creates the unique opportunity to examine urban source impacts in the absence of long-range pollution. The city is monocentric with a geographically small central business district (CBD) (approximately 5 km^2^) in a U-shaped basin ringed on three sides by ridges of up to 100 m elevation formed by extinct volcanoes, and with a harbor waterfront to the north. The Auckland CBD is home to 52,000 residents, with a population density of up to 10,000 km. With over 75,000 workers, the area hosts approximately 200,000 persons/day, on average [16]. The Auckland CBD contains a wide range of building morphologies and a dense emission source mix, including the vehicular and diesel sources common to western cities, a major port immediately adjacent, but few major industrial sources and no urban power plants. Thereby, Auckland offers a unique context to examine local impacts of multiple urban emissions sources, in a marine climate with little upwind emissions contribution, and where distinct patterns in local meteorology and topography may modify or intensify source-concentration relationships.

Despite the complexity of this setting, air pollution surveillance and regulatory decision-making for the CBD area, to date, has largely relied on data from a single monitoring station, operated by Auckland Council on Queen Street, a high-traffic road in the center of the CBD, and the city’s main commercial thoroughfare. It is normally interpreted as a ‘peak’ site, for purposes of New Zealand air quality regulations. Source apportionment analyses of data from this site have attributed PM_2.5_ to multiple sources, including motor vehicles (33–40% of PM_2.5_), marine aerosol (23%), biomass burning (14%), construction/crustal resuspension (11%), and ship sulphate (7%). Other small sources comprised less than 5% of the total [17]. Although these contributions differ substantially from those found at suburban regulatory sites [17], and a spatial saturation study of passive NO_2_ found a two-fold variance across the inner CBD, even between adjacent streets [18] no prior studies have examined how particle composition and source contributions might vary across the CBD.

Most source apportionment studies in Auckland have used back-trajectory modeling; this approach, when relying on data from one or a few monitoring points, is limited in its ability to identify spatial locations of sources, given limited information on source-to-receptor distance. As a result, there can be substantial confounding among spatially-dense sources in a similar direction from the monitor, or where the monitor may be obstructed by buildings or other impediments restricting airflow in any given direction. As a result, the specific locations and characteristics of key sulphate sources remain unclear in urban Auckland; although this PM component is often associated with ship emissions, it may include local (e.g., port activities) and distant (e.g., shipping lanes, volcanoes) natural and anthropogenic sources. Emissions from each of these sources, however, likely disperse differently, given differences in directionality, source height (e.g., elevated ship plumes), emissions characteristics (e.g., temperature), and complex terrain among volcanoes and within street canyons, giving rise to very different, but previously unobserved, fine-scale spatial patterns.

In this study, we aimed: (1) to document spatial variation in multiple pollutants across the complex Auckland CBD; to date, most air quality work in this area (including source apportionment) has relied on fixed regulatory monitors; less research has explored spatial variation. (2) To identify key sources potentially explaining spatial variation in pollution across the CBD, for source quantification and potential intervention. (3) To examine and demonstrate the utility of a combined spatial saturation/source tracer approach, leveraging spatial characteristics (e.g., variation in source distance/terrain) and source-specific elemental tracers to identify key sources.

To better disentangle the impact of multiple sources across this complex and densely-populated CBD, we designed a multi-pollutant saturation study, including both elemental and organic source tracers, focused on workweek hours (Monday–Friday, 7 am–7 pm), when the largest population may be exposed in the CBD. We identified tracers for key local sources of interest, and allocated sites to capture variability in those sources hypothesized to contribute most to spatial variation in pollution (i.e., traffic emissions, diesel (buses and trucks), proximity to port activity). We hypothesized that: (1) concentrations of PM_2.5_ and constituents may vary substantially, even across a small CBD, (2) that spatial patterns may differ for tracers of different sources, (3) that concentrations may vary by source proximity and density, and (4) that meteorological conditions may modify impacts of key sources (e.g., port) on observed concentrations and spatial patterns. 

## 2. Materials and Methods 

### 2.1. Study Domain Selection and Characterization

**Figure 1:**
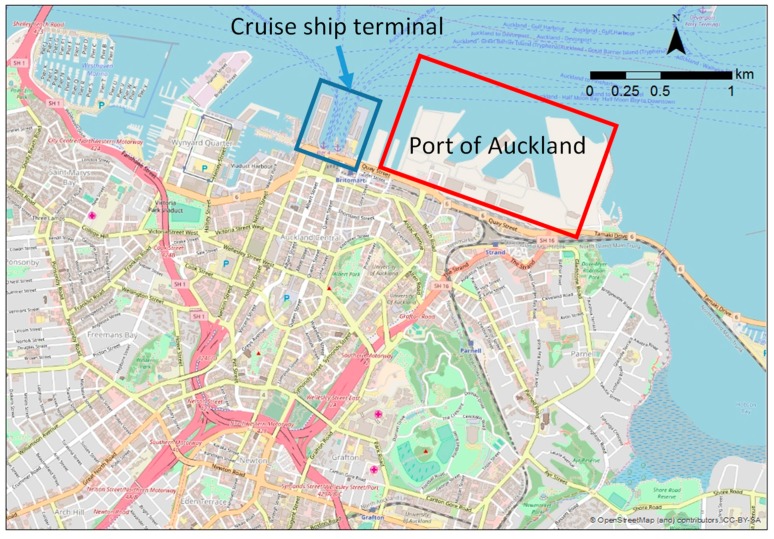
The Auckland central business district (CBD) is surrounded by motorways to the west, south, and east, and the waterfront to the north.

Auckland’s CBD is relatively compact, situated at sea level among extant volcanoes, and roughly circumscribed by major highways to the west, south, and east, and the waterfront to the north. The central area of the waterfront features a major transport interchange used by diesel buses, trains, ferries, and a cruise ship terminal (90 vessel-visits in 2015). The eastern end of the waterfront is dominated by the Port of Auckland (1558 vessel-visits in 2015) (Figure 1). We focused site selection on three key hypothesized sources: road traffic, diesel road traffic, and port-related emissions. 

To define our sampling domain, we fit a polygon in GIS that contained the CBD, major highways, and port, for a total study area of approximately five km^2^. We divided the study domain into a grid of 50 × 50 m cells (Figure S1), and characterized each cell by each of three key sources of interest (total traffic, diesel traffic, proximity to port) (Figure S2). Total traffic was estimated using annualized average daily weekday traffic, generated using the Auckland Regional Traffic model (data supplied courtesy of Auckland Council); we used a kernel density function to approximate emissions decay alongside roads, averaged the values from this surface within each 50 m^2^ grid cell, and dichotomized cells at the 70th percentile, to identify “low” and “high”-traffic cells. To calculate diesel (truck and bus) density, we multiplied traffic counts by the estimated percent heavy commercial vehicles on each road (from Auckland Regional Traffic model), applied a kernel density function, as previous, and dichotomized cells at the 70th percentile. Distance to port was estimated by fitting a polygon over the port area in GIS, identifying the centroid of the polygon, and calculating distance to this point from the centroid of each grid cell. Cells were dichotomized at the 20th percentile (383 m distance).

### 2.2. Site Selection

Due to equipment limitations and time constraints, sample size was limited to 12 spatially-distributed locations, plus two reference sites, over four weeks. We monitored two sites during every session, to assess temporal differences at locations of very different terrain within the CBD—one site was located in a street canyon in the valley (Albert St), and one exposed high on a hilltop in a city park (Albert Park). These sites have very different direct exposures to background (long-range) air pollution, and may reflect the range of impact that local meteorology may have on pollution measures at very different sites across this area. Comparing these two sites, concentrations were consistently higher on Albert St (Figure S3).

**Figure 2:**
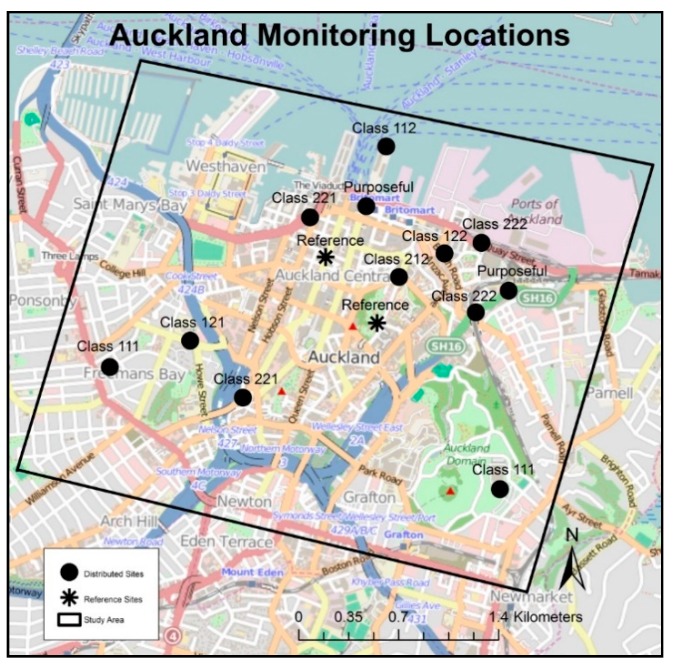
Distributed sampling sites across the study area. Classifications are, in order, traffic (1 = low, 2 = high), diesel (1 = low, 2 = high), and distance to port (1 = far, 2 = near).

To most efficiently capture spatial variation across the sources of interest, we cross-stratified each 50 m^2^ grid cell across our domain into eight classes, including all combinations of “high” and “low” density for each source. Stratified random sampling without replacement was used to select eight cells (one per strata), while ensuring reasonable spatial coverage. One additional site was selected from the hypothesized highest-exposure strata (high traffic—high diesel—near port), and one from the lowest-exposure strata (low traffic—low diesel—far from port). Two monitoring sites were selected near key points of interest (i.e., a central transportation hub, a heavy diesel-traffic location). Figure 2 depicts the final spatial allocation.

### 2.3. Sampling Pole Selection

To avoid contamination of quartz filters by off-gassing VOCs from treated wooden poles [19] we used only metal utility poles, selecting the one nearest to the centroid of each selected 50 × 50 m grid cell. We ensured there were no buildings, trees, overhanging branches, or other obstructions within three meters of each pole, and that each was directly accessible from the street. Latitude and longitude for each selected pole was identified in Google Earth. During site visits, field teams documented any temporary source activities (e.g., construction) not observed in GIS datasets. During sampling, no construction activity or other large confounding sources were observed near monitoring sites. 

### 2.4. Tracer Constituent Selection

We identified constituent tracers, in the elemental and organic fraction of PM_2.5_, based on a prior literature review [20,21,22,23,24,25,26] and prior source apportionment analyses in Auckland [17]. BC and all of the selected organic tracers (polycyclic aromatic hydrocarbons (PAHs) including fluoranthene and pyrene, hopanes, and steranes (i.e., cholestanes)) have been previously associated with diesel emissions [27]. Al and Ca have been associated with crustal resuspension from roadways and construction. NO_2_ and La have been associated with motor vehicle (gasoline) emissions [28,29]. Barium (Ba) and Antimony (Sb) have been used as tracers of brake and tire wear [30,31,32]. Ship emissions have been captured by vanadium (V) [33,34] and sulfur (S) [17]. Other oil burning sources have been captured by nickel (Ni) [33,34], and marine aerosols (sea salt) by sodium (Na) [17].

### 2.5. Sampling Instrumentation

Portable air sampling units [27] were used to collect samples of PM_2.5_, using Harvard Impactors (Air Diagnostics and Engineering, Inc., Harrison, ME, USA) on 37-mm Teflon™ filters (PTFE membrane, 2-µm pores, Pall Life Sciences). Battery-operated vacuum pumps (SKC, Inc., Eighty Four, PA, USA) were operated at a flow rate of 4.0 L/min, adjusted prior to deployment based on temperature forecasts; pre- and post-flow rates were determined to be within 5% of the target flow rate, then averaged to determine a mean temperature-corrected flow. A comparable co-located instrument—adapted to use a cyclone inlet for size selection, to avoid potential contamination of quartz filters by impactor grease—was used to collect PM_2.5_ samples for organic analysis, on pre-baked 37-mm quartz fiber filters (Pallflex Tissuquartz non-heat treated filters, Pall Life Sciences). A HOBO data logger was used to monitor both temperature and relative humidity at each site throughout sampling (Onset Computer Corporation). Instruments were housed inside weather-tight Pelican boxes mounted approximately three m above ground, near the breathing zone. Passive nitrogen dioxide (NO_2_) samples were collected using Ogawa badges (Ogawa & Co., Pompano Beach, FL, USA) in weatherized external shelters. Sampling instrumentation and protocols are further detailed in Tunno et al. 2016.

### 2.6. Analytic Procedures

Teflon™ filters were pre- and post-weighed using a Mettler Toledo ultramicrobalance (Model XP2U, Columbus, OH, USA) in a temperature- and humidity-controlled glovebox (PlasLabs Model 890 THC, Lansing, MI, USA) to determine total PM_2.5_ mass. An EEL43M Smokestain Reflectometer (Diffusion Systems, Ltd., London, UK) was used to measure BC absorbance (abs); this method relies on optical properties of the sample (reflection of light through the exposed filter) to estimate the concentration of light-absorbing carbon in a PM sample. To further detail the elemental composition of PM_2.5_, inductively-coupled plasma mass spectrometry (ICP-MS) was performed on Teflon™ filters according to documented protocols (ESS INO Method 400.4; EPA Method 1638) [35] by Wisconsin State Laboratory of Hygiene. Organic constituent concentrations were measured using thermal desorption gas-chromatography mass-spectrometry (TD-GCMS) on quartz fiber filters by Desert Research Institute (DRI) (Reno, NV, USA). For passive (24-h/day) NO_2_ concentrations, Ogawa passive badges were analyzed by water-based extraction and spectrophotometry on a Thermo Scientific Evolution 60S UV-Visible Spectrophotometer, Waltham, MA, USA).

### 2.7. Filter Handling Protocols

Because of the relative instability and volatility of organic compounds, we developed and refined laboratory and field protocols to minimize contamination. For organics sampling, we used pure quartz filters with no binder or glass fibers, placed into porcelain dishes using Teflon-coated tweezers, and baked for four hours at 900 °C prior to deployment (Thermo Scientific Thermolyne oven, Waltham, MA, USA), to remove any trace organics. All cyclone accessories were cleaned using methanol in a fume hood; we methanol-cleaned and foil-covered all sampler inlets throughout lab and field operations prior to deployments, to further reduce contamination. During retrieval, the quartz filter was removed from the cyclone sampler, stored in a sterile petri dish, and placed in an insulated box with ice packs. In the lab, quartz filters were kept inside the petri dishes, wrapped in aluminum foil, and stored at −20 °C until shipped on ice for analysis.

### 2.8. Sampling Intervals

Sampling was performed over four weeks from 6 April to 2 May 2014. To focus on work-hour exposures in the CBD, samplers were programmed using a chrontroller (ChronTrol Corporation, San Diego, CA) to run over five days (7 am–7 pm, Monday through Friday). NO_2_ passive badges collected samples continuously throughout the five days. 

### 2.9. Temporal Allocation, Reference Monitors, and Temporal Adjustment

The distributed sites for both the PM_2.5_ and gas sampling campaigns were randomly allocated across the four sampling sessions, ensuring variation across space and source categories every week. Because different sites were sampled each week, we used the temporal data collected every session at one “reference” site in a city park (Albert Park), set back from combustion sources. This method has been successfully used in other urban air pollution campaigns, and it was assumed that the “temporal” portion of pollution variance (due to long-range transport and meteorology) is constant across the city. To test this assumption, given the complicated meteorology and terrain of Auckland, we collected measures at two very different sites during all four weeks of the study, and compared these to assess the difference in that “temporal” component of concentrations between sites. Because these two sites did differ in observed trends, we opted to use the site less impacted by local sources and pollution entrapment (i.e., street canyons) as our “reference” site. 

For each of the spatially-distributed sites, we calculated a temporally-adjusted concentration, by dividing observed concentrations by the session-specific concentration at this Albert Park “reference” site, then multiplying by the seasonal-average concentration in the park, as in Shmool et al. 2014 [36]. We averaged the four concentration measures from the reference site, and included it in the spatial analysis, for a total of 14 locations. 

### 2.10. Quality Assurance/Quality Control

We collected four laboratory blanks and four field blanks, for all pollutants every session, to identify any potential sources of contamination. Each sampling session, we co-located four monitors (two using Teflon™, and two using quartz filters) at one randomly-selected site, to examine reproducibility. All filters were visually inspected for potential tears and wetness, before and after all deployments. All Teflon™ and quartz samples (100%) met acceptable pre- and post-collection flow rates, within 5% of 4 LPM. Co-located measures of PM_2.5_, BC, and NO_2_ were highly correlated (r = 0.91 to 0.99) across monitoring locations. Field blanks for all pollutants were near zero for all four sampling sessions; session-specific blanks were used correct observed concentrations at distributed sites. 

### 2.11. Data Analysis

We identified no statistical outliers (defined as mean ± 3 SD) and calculated descriptive statistics for PM_2.5_, BC, NO_2_, and all elemental and organic constituents (Table 1). We then examined correlations among pollutants, and between pollutants and source density indicators, using Spearman correlations (due to our small sample size and potential non-normal distributions), scatterplots, and least-squares regression. Maps for all pollutants were produced, and visually examined and compared, using ArcGIS v 10.4 (Redlands, CA, USA). Statistical analyses were performed in SAS, v 9.4 (Cary, NC, USA). 

### 2.12. Meteorological Data

Wind speed and direction data was obtained from a permanent pair of anemometers installed at 318 m above sea level on the tallest building in the central CBD. Although wind speed at this site over-estimates wind speed at ground level, it provides for internal comparison between sampling sessions. 

## 3. Results

Data completeness was 98%, with no statistical outliers (outside of mean ± 3 × standard deviation) for any pollutant. Co-located measurements indicated strong reproducibility for all pollutants. One site was eliminated from organics analysis, due to a quartz filter was found wet upon retrieval. Likewise, NO_2_ samples were lost at two sites (on Wellington St and Tyler St), due to heavy rain, and thus data was 93% complete.

### 3.1. Summary Statistics

Though concentrations were low relative to other major urban areas worldwide, the range in concentrations for almost all pollutants was substantial, even across a small CBD. PM_2.5_ concentrations averaged 7.0 µg/m^3^ (SD = 2.2 µg/m^3^) across the 14 sites, but ranged from 4.5 to 11.3 µg/m^3^. For NO_2_, we observed an average concentration of 14.6 ppb (SD = 4.7 ppb), ranging from 8.3 to 23.7 ppb.

Among all constituents, we found the highest concentrations for sodium (Na) with a mean of 197.8 ng/m^3^ (SD = 163.1 ng/m^3^), as may be expected in a marine climate, and consistent with findings from a previous source apportionment study in Auckland [17] The greatest range in concentrations was observed for vanadium (V), a commonly-used tracer of shipboard oil burning emissions, which varied more than 40-fold across this small area (1.2 to 48.1 ng/m^3^). 

### 3.2. Common Spatial Variation and Correlations Among Pollutants

**Figure 3:**
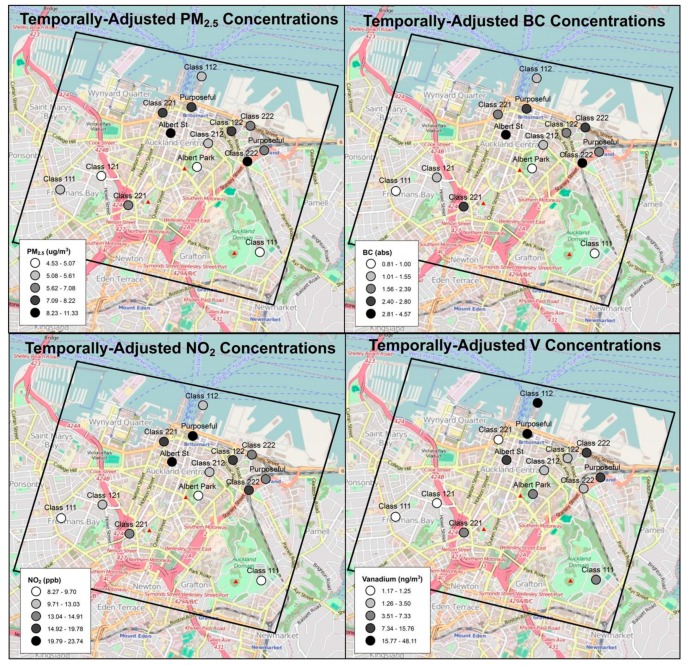
Temporally-adjusted PM_2.5_, BC, NO_2_, and V concentrations (in quintiles) across 14 monitoring sites.

As noted above, PM_2.5_ concentration varied 2.5-fold, even across this small CBD as shown in Figure 3. As expected, PM_2.5_ concentrations were positively associated with traffic and diesel, and negatively associated with distance from port (Figures S4 and S5). 

Concentrations of constituent source tracers also varied substantially, and spatial patterns differed by pollutant; however, many constituents were highly correlated, consistent with hypothesized common sources (Table 2). BC, for example, a commonly-used tracer for diesel, varied 5.6-fold (range = 0.8 to 4.6 abs), and was highly correlated with PM_2.5_ (r = 0.97) with a least-squares regression intercept of 3.1 µg m^−3^, indicating a consistent background PM_2.5_ concentration of this magnitude derived from non-local sources, including sea salt. NO_2_ varied from 8.3 to 23.7 ppb, and was correlated with both BC and PM_2.5_ (r = 0.90 and 0.88, respectively). 

PM_2.5_ and BC were also highly correlated with all organic constituent concentrations (r > 0.70 in all cases). Hopanes and steranes were highly correlated (r = 0.94), though less so with the other organic tracers or with lanthanum (all tracers for motor vehicles/diesel, and r = 0.52–0.66 in all cases). Flouranthene and pyrene very highly correlated with total PAHs (r > 0.95, as expected, given that total PAHs include both). Barium and antimony (both indicators of brake/tire wear) were highly correlated (r = 0.91). Aluminum and calcium (soil resuspension) were correlated with one another (r = 0.56). Although not correlated with the traffic-related markers described above, sodium (sea salt), nickel (oil burning), and sulfur (sulphate) were all correlated (r = 0.65–0.79 in all cases). Both nickel and sulfur were correlated with vanadium (all ports emissions tracers, r = 0.56–0.76).

A visual inspection of the data indicates higher concentrations of PM_2.5_ and several constituents (i.e., vanadium, hopanes, steranes) nearer to the port and at locations with higher traffic and diesel density (Figure 3). Maps broadly supported hypothesized sources for each pollutant, and common spatial patterns were observed among correlated pollutants. 

### 3.3. Wind Direction and Implications for Spatial Patterns

**Figure 4:**
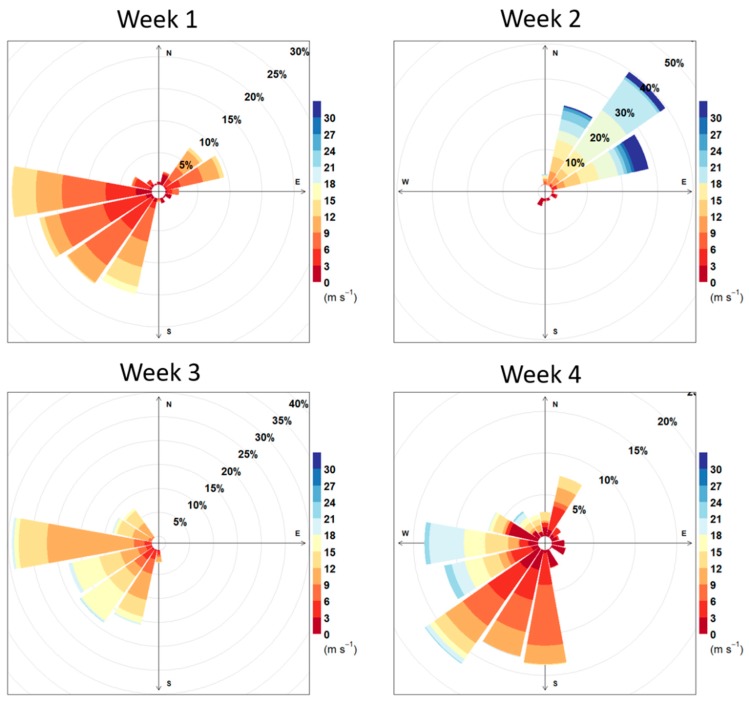
Wind roses for the four sampling sessions. Data is collected from 318 m above sea level; as such, wind speeds are generally higher than at ground level.

Of the four weekly sampling sessions, Session 2 presented substantially different meteorological conditions (i.e., wind direction) relative to the other three (see Figure 4). Notably, session 2 was dominated by north-easterly winds, including an ~12 h period of high winds associated with a storm, in contrast to the predominant south-westerly winds observed in sessions 1, 3 and 4. 

**Figure 5:**
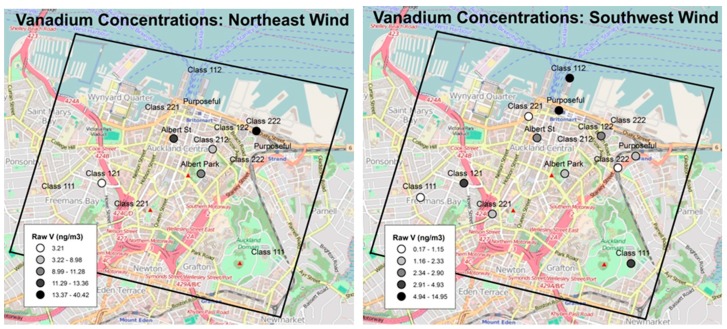
Maps of elevated vanadium concentrations were detected during Session 2 (predominant winds from north-east), versus lower concentrations during Sessions 1, 3, and 4 (winds from southwest). Note that maps are on different scales for visualization purposes.

**Figure 6:**
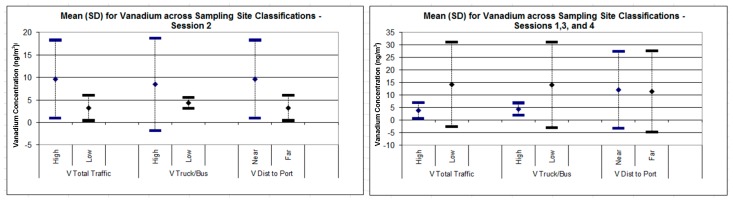
Boxplots of vanadium concentrations versus high/low source density, for Session 2 (northeasterly winds) versus Sessions 1, 3, and 4 (southwesterly winds).

During Session 2, relative to other sessions, concentrations of ports emissions and marine source tracers (e.g., V, Ni, Na, S) were all elevated, on average, from 1.5- to five-fold, across all sites (Figure 5). Vanadium concentrations were far higher, on average, in the high-source-density categories only during Session 2 (Figure 6). In contrast, elements not likely related to ports emissions (e.g., Fe and Al, potential markers of diesel, soil/road dust resuspension from traffic and NO_2_) fell substantially. 

Two outliers were sampled during Session 1 (Britomart (the central “Purposeful” site in Figure 2), V = 48.1 ng/m^3^) and Session 3 (Queens Wharf (the upper “Class 112” site in Figure 2), V = 35.0 ng/m^3^). Both sites are alongside the busy commuter ferry terminal and cruise ship docks, and thus heavily impacted by marine traffic emissions during any session, regardless of wind direction. Removing these two outliers produces a mean V concentration of 4.53 ng/m^3^ (SD = 6.27) during sessions 1, 3, and 4, versus 9.63 (SD = 3.23), a two-fold difference, during Session 2.

Comparing PM_2.5_ versus session-average windspeed at the two sites monitored throughout the study, PM_2.5_ generally decreased with higher windspeeds, as expected. However, spatial differences between these sites were substantial and consistent throughout the study, with higher concentrations on Albert St (heavily-trafficked street canyon) than in Albert Park (elevated green park) (Figure S6).

## 4. Discussion

Given growing evidence of the impact of chronic urban air pollution exposures on respiratory, cardiovascular, and other health outcomes [5,6,7,8,9,10,11,12], there is now a rich literature on intra-urban variation in concentrations of multiple pollutants and source tracers. Many of these exposure assessment studies were developed to improve air pollution exposure assessment for epidemiology [15,37], or to support local policy and urban planning purposes [13,38]. Most such studies, however, have been performed in North America or Europe, and none within an urban environment isolated by thousands of kilometers of ocean. In Auckland, New Zealand’s largest city, a high density of vehicular traffic, trucks, buses, and a large immediately-adjacent port, together produce substantial spatial variation in pollution, even across a small (5 km^2^) CBD. Because the Auckland CBD is the mostly densely populated area in New Zealand, environmental conditions including air quality in this area are among the measurable targets of Auckland Council’s Auckland Plan 2050, designed to balance rapid population and economic growth with minimizing environmental degradation and related human health effects [39].

Given New Zealand’s isolated position in the Southern Hemisphere, Auckland offers the unique opportunity to examine urban source impacts in the absence of long-range air pollution. Given the low background, we observed lower absolute concentrations than in other western cities of comparable size; however, the relative differences in concentrations between sites are more pronounced, as locally-generated emissions constitute a greater proportion of ambient concentrations. Thus, despite our small sample size, we found substantial spatial variation in PM_2.5_, elemental constituents, and organic compounds, not previously observed within the Auckland CBD, which were associated with varying source densities and source proximity, modified by wind direction. In general, we found higher concentrations in street canyons and in areas of denser traffic and/or near the port, including at one site on the principal truck route to/from the Port, adjacent to a railway for diesel-powered passenger trains.

Though the concentrations observed in Auckland are, in many cases, lower than those observed in North American and European cities, the concentrations of some source-specific constituents are particularly high. For example, PM_2.5_ in Auckland averaged only 7.0 (2.2) µg/m^3^, lower than any of the 20 European cities monitored by the ESCAPE consortium [40]. Only five ESCAPE cities, however, had any observed V measurements, at any sites, above 5.0 ng/m^3^ [15]; in contrast, more than half of our sites had V concentrations above 5.0 ng/m^3^, with an overall average of 10.4 (SD = 13.5) ng/m^3^. This notably large proportion of variation in PM_2.5_ attributable to a constituent indicative of a key local source (i.e., ports) supports our expectation that a relatively large proportion of PM_2.5_ in Auckland is local in origin.

The chance occurrence of a distinct and sustained change in wind direction during Session 2 provided insight into the impact of port-related emissions and the role of meteorology in modifying spatial patterns. During three of our four sampling weeks, predominant winds were from the southwest, and thus, the CBD was downwind of other Auckland neighborhoods but upwind of the Port. During Session 2, however, winds were predominantly north/northeasterly, with above-average wind speeds). During this session, we generally observed lower pollution concentrations; however, substantially greater impacts of ports-related emissions on observed concentration gradients and spatial patterns, particularly for hypothesized tracers of marine and ports-related sources [41]. This finding suggests that activities associated with the Port of Auckland are a significant local emission source, albeit usually downwind of the city center, due to Auckland’s prevailing south-westerly winds.

Our results exemplify a methodological challenge related to the siting of reference monitors and temporal adjustment for spatial analysis, in areas of frequently-shifting meteorological paradigms, complex terrain which re-directs airflows, and/or concentrations below LOD for key pollutants and constituents of interest. Many prior spatial saturation studies have measured at a few fixed “reference” sites throughout the study, and used these measures to temporally-adjust measures collected at short-term distributed sites, to account for temporal differences in meteorology and long-range transport [13,21]. This method works well in areas with reasonably stable spatial patterns in concentrations—where topography and meteorological variations across sessions do not alter the overall spatial pattern in concentrations. However, emissions, dispersion, and advection can be significantly constrained and defined by local meteorology and topography, particularly in areas of high buildings density or complex terrain. Under these conditions, appropriate “upwind” locations may be difficult to identify, and potential reference sites within the study may be overly impacted by local sources, or by fine-scale re-directions in airflow. In this case, meteorological conditions, especially wind direction, substantially influenced source impacts at various points in time, shaping and re-shaping observed concentration patterns across the CBD, with notable differences across constituents. For purposes of this study, we selected Albert Park as a reference site, due to its location within the CBD, relative openness (i.e., not subject to street canyon effects), and relatively low exposure to near-field sources, as it is more than 150 m from any road. This site, however, is at a higher elevation than much of the CBD, and therefore may be more exposed to ship plume emissions (from elevated ship stacks) than are other sites—an effect which may be highly dependent on wind direction.

A further limitation of our study, related to the small sample size and limited number of sites measured each session, was that we could not capture variance in every variable which may influence spatial variance in concentrations—including cooking emissions (e.g., food outlets), roadway gradient and density of intersections/idling, diesel bus emissions, trains, ferries, and cruise ships (90 cruise ships docked in Auckland in 2014, but only one during our study). Additional variables influencing source-receptor relationships could include ground elevation (ranging from 0 to ~100 m across the study area) and building height (which is log-normally distributed, with only 6% of buildings in the CBD over 40 m tall).

Concentrations observed in this study cannot be compared to New Zealand air quality standards, as the country currently has no ambient standard for PM_2.5_. Nor should concentrations be directly compared to US or EU standards, as the measures reported here are neither 24-h nor annual-average concentrations, which are the basis for those standards. Nevertheless, PM_2.5_ has been monitored routinely for over a decade by Auckland Council on Queen Street, a high-traffic street-canyon road in the center of our study area, and the city’s main commercial thoroughfare, chosen as a ‘peak’ site, for purposes of New Zealand air quality regulations. PM_2.5_ concentrations measured at this site during our campaign averaged 7.1 µg/m^3^—comparable to the mean of our sampling sites (7.0 µg/m^3^), but lower than concentrations at four sites, including Albert Street (11.3 µg/m^3^), only one block away.

Despite the limitations of our small sample size, our study was successful in identifying and capturing spatial variation in multiple pollutants during workweek hours (Monday–Friday, 7 am–7 pm), when the largest population may be exposed, across the Auckland CBD. The differing spatial patterns in elemental and organic constituent source tracers revealed distinct impacts of key local sources—particularly the port and diesel traffic—and these fine-scale spatial patterns have not been previously explored in Auckland, a relatively isolated western city where source impacts may be examined in the absence of substantial background pollution. Finally, stratifying sampling weeks by predominant meteorological conditions, particularly windspeed, revealed substantial differences in observed spatial patterns; this meteorological influence of source-specific impacts on spatial variation in exposures within dense urban cores merits further study.

## 5. Conclusions

Our monitoring campaign successfully captured spatial variability in locally-derived PM_2.5_ during workweek hours (Monday–Friday, 7 am–7 pm) across a small but densely-populated CBD. While PM_2.5_ concentrations varied 2.5-fold across the area, source-specific constituents varied more dramatically; for example, BC varied more than five-fold, with higher concentrations in areas of greater traffic and diesel activity, and V varied more than 40-fold, with higher concentrations nearer the port. However, the overall spatial patterns and observed source impacts varied with meteorological conditions, especially wind direction. Even in the absence of substantial background pollution, these complex interactions among meteorology, multiple sources, and complex topography across dense urban cores–and the consequent impacts for population exposures and public health—merit further study.

## Figures and Tables

**Table 1:** Temporally-adjusted pollutant concentrations across sites. Organic constituents were not blank-corrected because no concentrations > LOD were detected at Albert Park reference site, and thus temporal adjustment could not be performed. The sample size (*n*) for organics is 13, as one sample was lost due to wet filters. PAH: polycyclic aromatic hydrocarbon.

Pollutant	*n*	Mean (SD)	Median	Min	Max
PM_2.5_ (µg/m^3^)	14	7.0 (2.2)	6.7	4.5	11.3
BC (abs)	14	2.2 (1.2)	2.1	0.81	4.6
NO_2_ (ppb)	14	14.6 (4.7)	14.3	8.3	23.7
Elemental constituents (ng/m^3^)					
Al	14	7.8 (7.2)	4.8	1.05	25.2
Ba	14	0.89 (0.96)	0.69	<0.001	3.5
Ca	14	34.9 (12.6)	35.1	11.0	50.8
Na	14	438.0 (109.3)	434.3	258.7	640.1
Ni	14	7.7 (15.2)	1.4	<0.001	58.3
S	14	197.8 (95.8)	163.1	59.9	409.7
Sb	14	1.6 (1.3)	1.5	0.32	5.4
V	14	10.4 (14.0)	5.3	1.2	48.1
Organic constituents (ng/m^3^)					
PAHs:					
Fluoranthene	13	0.06 (0.05)	0.05	0	0.14
Pyrene	13	0.12 (0.11)	0.08	0	0.35
Total PAHs (fluoranthene + pyrene)	13	0.18 (0.16)	0.13	0	0.50
Hopanes and Steranes					
Total hopanes	13	0.49 (0.27)	0.39	0.20	1.20
Total steranes	13	0.58 (0.77)	0.27	0	2.71

**Table 2:** Spearman correlations for concentrations across sites, and hypothesized sources. Total PAHs includes fluoranthene and pyrene.

	PM_2.5_	BC	NO_2_	Al	Ba	Ca	La	Na	Ni	S	Sb	V	Fluor-	Pyrene	Total PAHs	Total Hopanes	Total Steranes	Hypothesized Sources
PM_2.5_																		
BC	**0.97**																	Diesel
NO_2_	**0.81**	**0.92**																Traffic
Al	0.17	0.18	**0.71**															Soil/resuspension
Ba	0.05	0.17	0.10	0.14														Brake/tire wear
Ca	**0.52**	0.40	0.47	**0.56**	−0.08													Soil/resuspension
La	−0.12	−0.05	**0.51**	0.00	−0.29	−0.05												Motor vehicles
Na	0.11	0.07	−0.32	0.37	−0.12	**0.71**	0.05											Sea salt
Ni	−0.02	−0.07	−0.26	−0.19	−0.24	0.25	−0.13	**0.65**										Oil burning
S	0.14	0.13	0.11	0.30	−0.15	**0.54**	−0.05	**0.79**	**0.76**									Sulphate
Sb	0.01	0.19	0.46	0.02	**0.91**	−0.25	0.02	−0.15	−0.17	−0.09								Brake/tire wear
V	0.06	0.05	−0.20	−0.09	−0.09	0.04	−0.15	0.28	**0.56**	**0.58**								Ship emissions
Fluoranthene	**0.80**	**0.70**	**0.84**	0.05	−0.10	0.48	**0.52**	0.03	−0.04	−0.10	−0.15	−0.08						Diesel
Pyrene	**0.89**	**0.82**	**0.91**	0.08	−0.03	0.46	**0.52**	0.01	−0.10	−0.05	−0.07	0.02	**0.95**					Diesel
Total PAHs	**0.87**	**0.79**	**0.89**	0.07	−0.05	0.47	**0.53**	0.02	−0.08	−0.07	−0.09	−0.01	**0.98**	1.00				Diesel
Total Hopanes	**0.77**	**0.77**	0.42	0.07	−0.31	0.33	**0.58**	0.03	0.02	0.10	−0.22	−0.14	**0.64**	**0.66**	**0.66**			Traffic
Total Steranes	**0.74**	**0.76**	0.26	0.01	−0.27	0.34	**0.59**	0.11	0.01	0.18	−0.18	−0.16	**0.52**	**0.58**	**0.56**	**0.94**		Traffic

Correlations > 0.50 displayed in bold.

## Supplementary Materials

The following are available online at https://www.mdpi.com/1660-4601/16/9/1567/s1, Figure S1: 50 × 50 m grid used for site classification in GIS, Figure S2: Source classification map used for site selection. Numerical class categories refer to (in order): Total traffic density, truck density, distance to port (1 = low, 2 = high, in all cases), Figure S3: Summary of data from Albert St and Albert Park sites, monitored during all four sessions, Figure S4: Scatterplots of temporally-adjusted PM_2.5_ concentrations versus source classes, Figure S5: Boxplots of PM_2.5_ and BC concentrations by source category (Total traffic density, truck density, distance to port). In all cases, we found non-normal distributions in concentrations, and slightly higher average concentrations in the high-source category, Figure S6: PM_2.5_ concentration versus windspeed, across sessions, at two reference sites (Albert St is a low-elevation street canyon in the center of CBD; Albert Park is a high-elevation green park adjacent to the CBD).
