# Supplementary material for: Assessment of Spatial Variability across Multiple Pollutants in Auckland, New Zealand
Source: Int J Environ Res Public Health. 2019 May 5;16(9):1567. doi: 10.3390/ijerph16091567 (PMC6539388; doi:10.3390/ijerph16091567)
Supplement: Supplementary file 1 [file ijerph-16-01567-s001.pdf]

Supplemental Material:

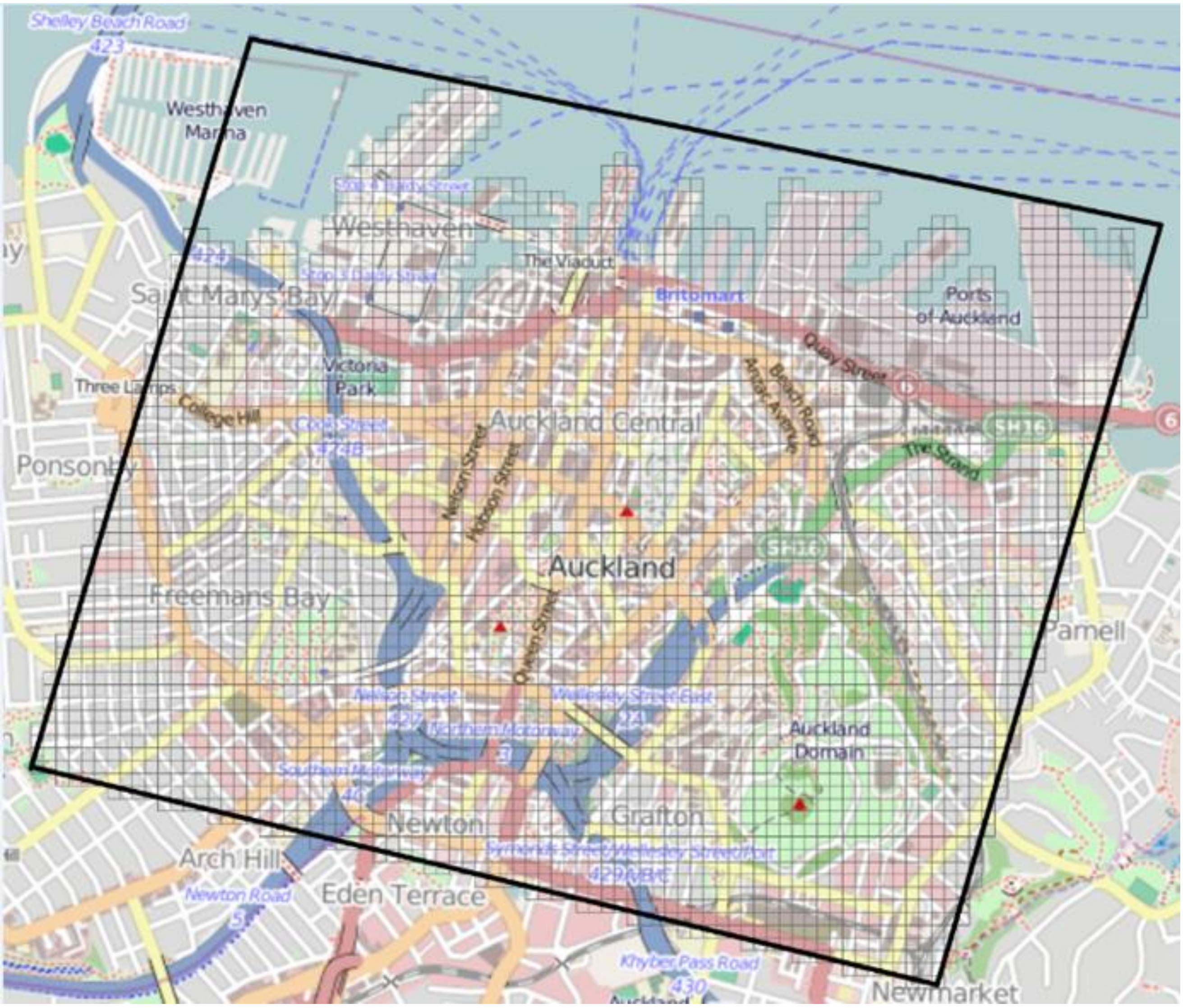

Figure S1. 50 × 50 m grid used for site classification in GIS.

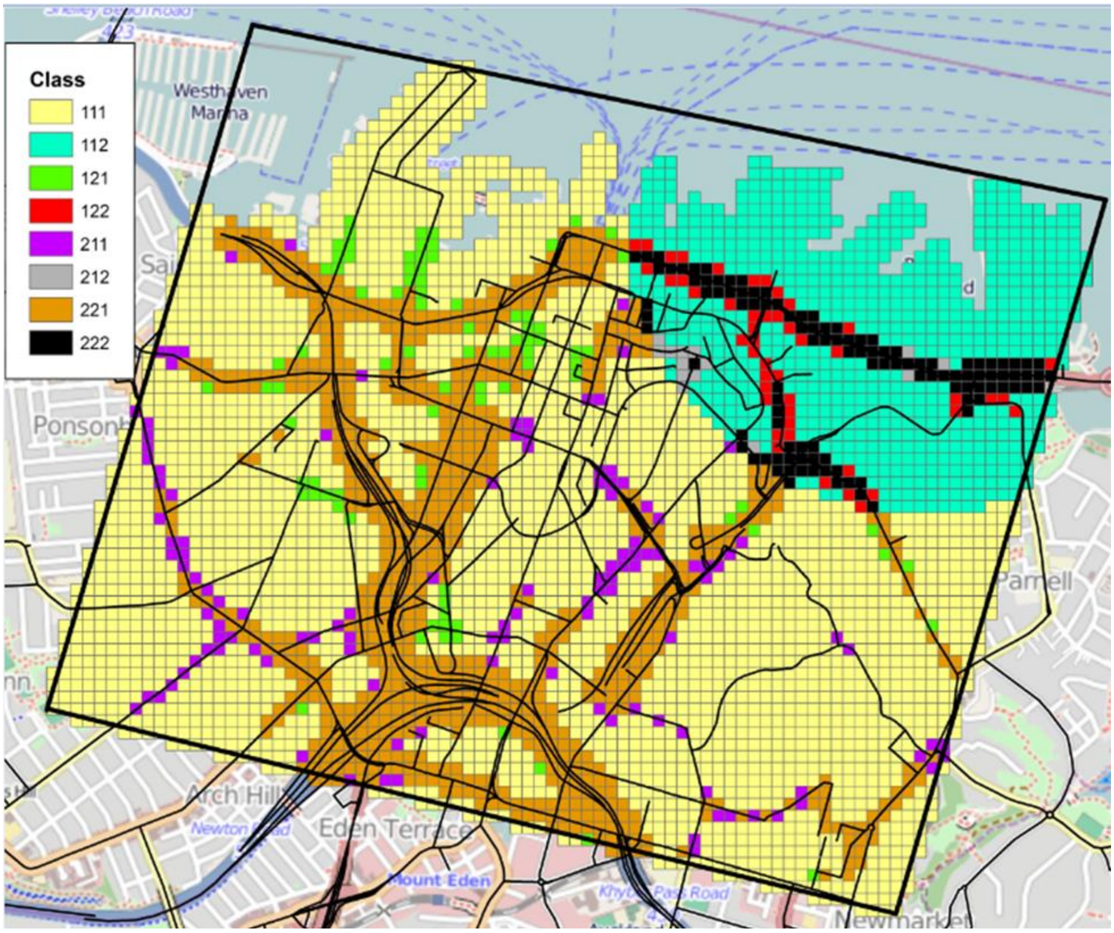

Figure S2. Source classification map used for site selection. Numerical class categories refer to (in order): Total traffic density, truck density, distance to port (1 = low, 2 = high, in all cases).

Session	Albert St	Albert Park
1	14.00	5.33
2	8.05	5.29
3	10.12	3.48
4	13.13	4.88
average	11.33	4.75
overall_avg	8.04	
		0.216754054

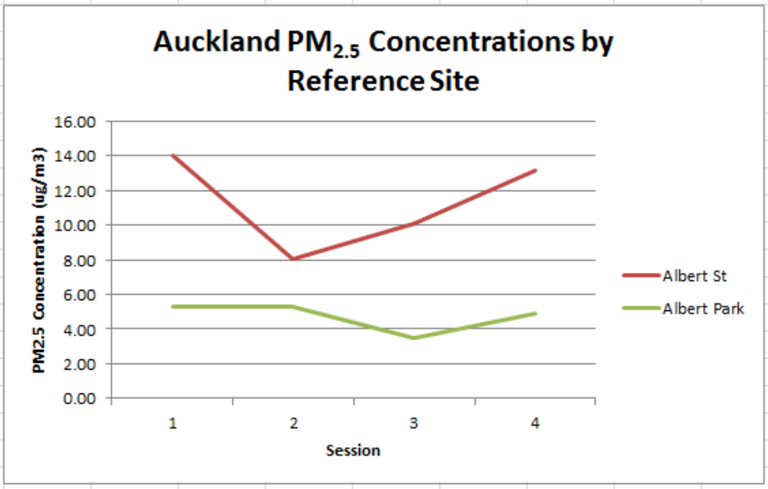

Figure S3. Summary of data from Albert St and Albert Park sites, monitored during all four sessions.

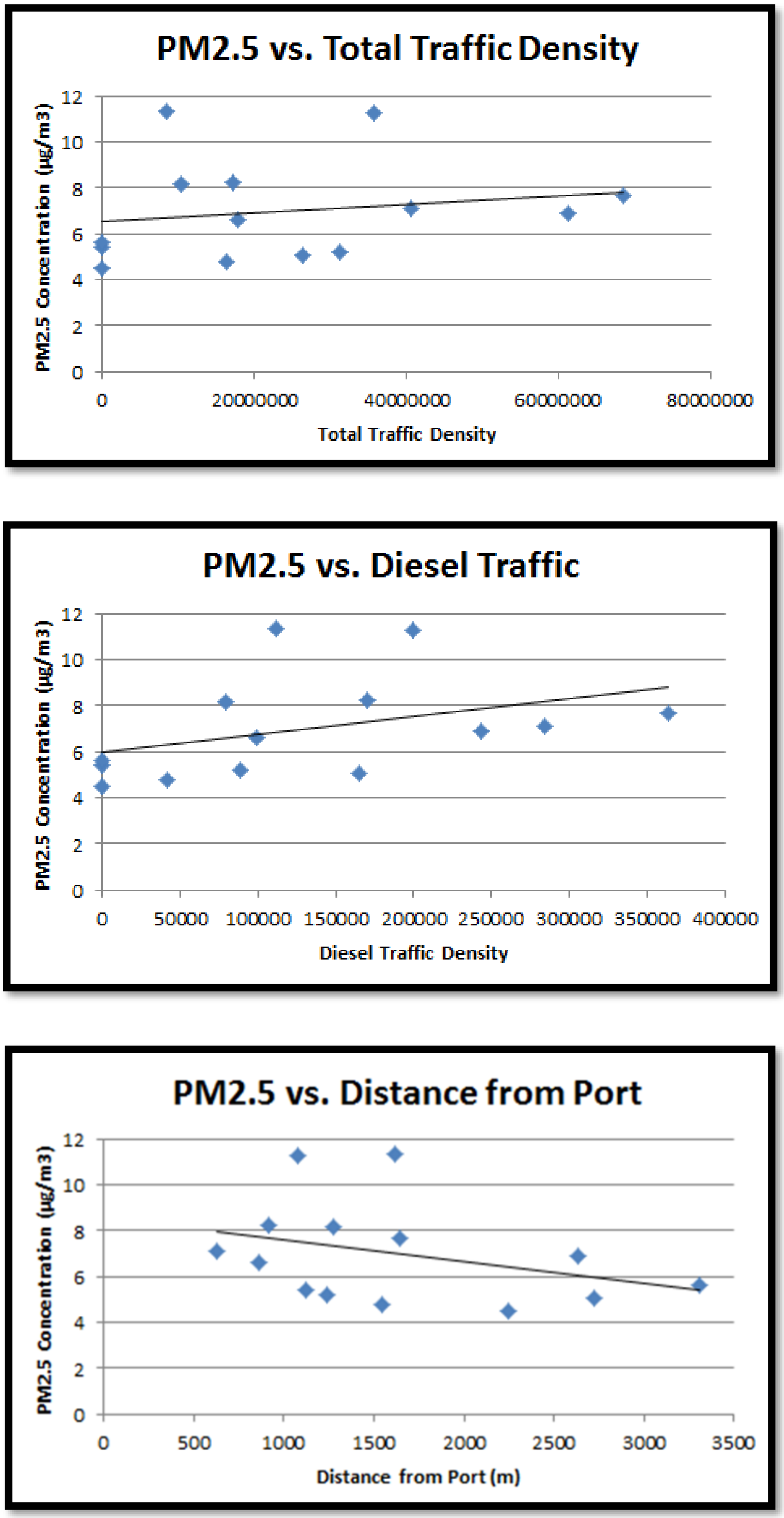

Figure S4. Scatterplots of temporally-adjusted $PM_{2.5}$ concentrations vs. source classes.

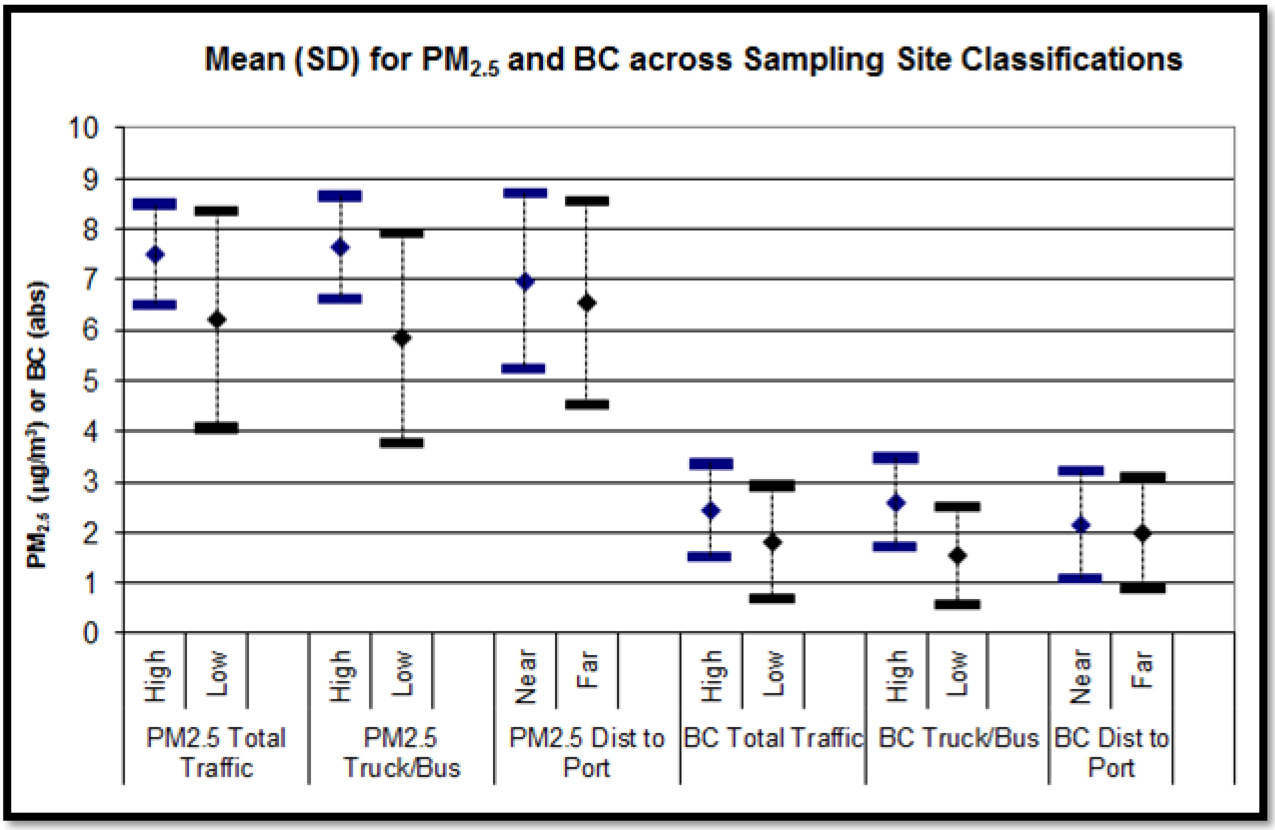

Figure S5. Boxplots of $PM_{2.5}$ and BC concentrations by source category (Total traffic density, truck density, distance to port). In all cases, we found non-normal distributions in concentrations, and slightly higher average concentrations in the high-source category.

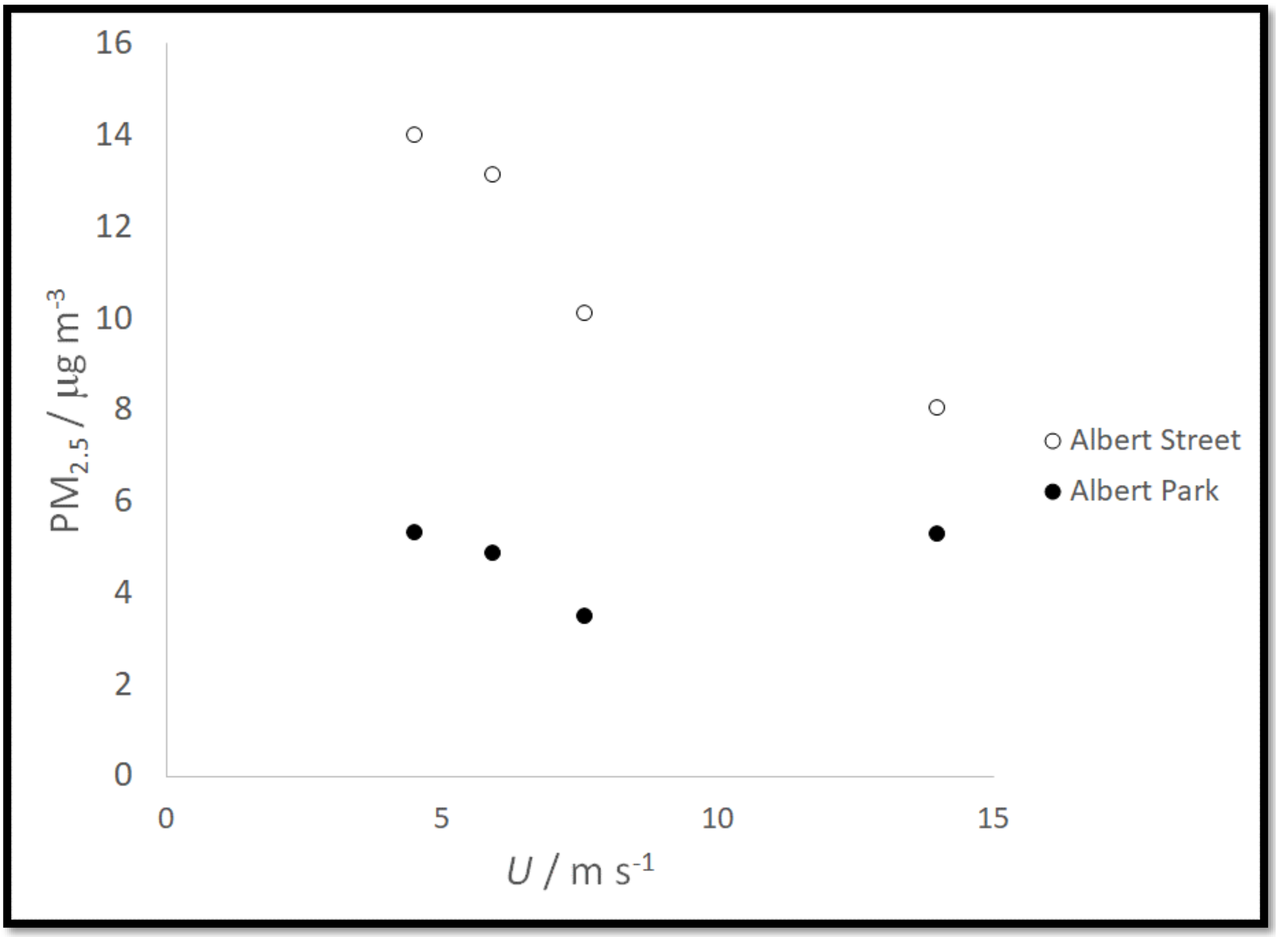

Figure S6. $PM_{2.5}$ concentration vs. windspeed, across sessions, at two reference sites (Albert St is a low-elevation street canyon in the center of CBD; Albert Park is a high-elevation green park adjacent to the CBD).
